# The private sector market for malaria rapid diagnostic tests in Nigeria: results of the 2018 market survey

**DOI:** 10.1186/s12936-022-04209-3

**Published:** 2022-06-16

**Authors:** Hannah M. Edwards, Rubaiyath Sarwar, Parvez Mahmud, Shekarau Emmanuel, Kolawole Maxwell, James K. Tibenderana

**Affiliations:** 1 Malaria Consortium Headquarters, 244-254 Cambridge Heath Rd, London, E2 9DA UK; 2 Innovision Consulting Private Limited, Level 3 & 4 House 26 Road 6 Baridhara J Block Pragati Sarani, Dhaka, 1212 Bangladesh; 3 Case Management Branch, National Malaria Elimination Programme, First Floor, Abia House, Central Business District, Abuja, Nigeria; 4 Malaria Consortium Nigeria, 33 Pope John Paul Street, Off Gana Street, Maitama, Abuja, FCT Nigeria

**Keywords:** Private sector healthcare, Case management, Diagnostics, Health economics, Malaria control, Informal health workers, Private sector engagement, Targeted subsidy, Private sector co-payment mechanism

## Abstract

**Background:**

To avoid misuse of anti-malarials, correct diagnosis of fever prior to drug prescription is essential. Presumptive treatment in the private healthcare sector is a concern in Nigeria, where availability of affordable artemisinin-based combination therapy (ACT) is high following the implementation of subsidy schemes from 2010 to 2017. Similar subsidies have not, however, been implemented for malaria rapid diagnostic tests (RDTs). A market survey in 2018 predominantly designed to assess the ACT market in the private sector also collected data related to RDTs, results of which are presented herein.

**Methods:**

A 2018 market survey consisted of (i) an outlet survey targeting private pharmacies and Proprietary and Patent Medicine Vendors (PPMVs) across different regions of Nigeria to assess supply-side market factors related to availability of RDTs (defined as having stock available for purchase at the time of the survey) and (ii) a household survey to determine demand-side factors related to knowledge of RDTs, healthcare-seeking practices and affordability.

**Results:**

Availability of RDTs at the time of the survey was low in both outlet types and significantly lower in PPMVs (22.1%, 95% CI) among pharmacies versus (13.6%, 95% CI) among PPMVs (p < 0.01). Reasons for not restocking RDTs included low demand and no supply. The majority of households diagnose malaria based on experience, while one-third would visit a PPMV or pharmacy. Half of households had heard of RDTs (48.4%) and 38.6% thought they were affordable.

**Conclusions:**

Low availability of RDTs among PPMVs and pharmacies may be attributed to lack of demand, supply-side issues and cost. Increasing household knowledge of RDTs may aid increasing demand, while subsidized RDTs may address supply and price issues. Addressing the deficit in RDT provision is important for targeting of ACT medicines.

## Background

The private, for-profit healthcare sector plays a significant role in malaria case management in Nigeria [1–3]. Since this market can be difficult to regulate and monitor, there are often concerns about whether consumers have access to appropriate, high quality and affordable diagnostics and treatments in line with current recommended guidelines [4]. To address financial barriers to accessibility of malaria treatment, subsidy schemes known as the Affordable Medicines Facility—malaria (AMFm) and, later, the Private Sector Co-payment Mechanism (PSCM) were in place in Nigeria between 2010 and 2017 to subsidize the cost of quality-assured artemisinin-based combination therapy (QA-ACT) in the private sector. A market survey was conducted in 2018 at the termination of the PSCM to assess the state of both demand and supply side market factors related to malaria diagnostics and treatments among Proprietary and Patent Medicine Vendors (PPMVs) and pharmacies in the private sector. A recent study presented the results of this survey in relation to ACT and found both availability and affordability of ACT medicines in Nigeria increased, particularly among PPMVs, during the AMFm-PSCM period [1, 5]. Interestingly, these improvements were not limited to just the subsidized ACT brands; in response to increased market competition, non-subsidized ACT brands had reduced prices and increased their availability and market share [5].

Although ensuring access to QA-ACT is integral to successful case management, proper diagnosis using QA diagnostics before treatment is also essential. A study of registered private hospitals and clinics in Nigeria in 2014 found high use of malaria diagnostics mostly via microscopy [6]. Microscopy is unlikely to be available in many private outlets or in very rural communities [1]. Easy-to-use malaria rapid diagnostic tests (RDTs) present an effective option in these settings but correct RDT use appears low and presumptive treatment of fever cases with anti-malarials remains high, particularly in the private healthcare sector [7–11]. Despite this, engagement with the private sector to promote RDT availability and use has been limited; a review found just 12 studies where RDTs had been introduced to drug shops or pharmacies and these were predominantly small-scale pilots [12].

This paper presents results of the 2018 market survey in relation to supply of and demand for RDTs from these outlets. As an integral part of malaria case management but not subject to any subsidy scheme, assessment of the malaria RDT market may act as an important comparator to that of ACT medicines.

## Methods

In 2018, both an outlet survey and a household survey were conducted to assess the ACT and RDT private sector markets in Nigeria. Full methods are presented in Edwards et al. [5]. Briefly, 321 pharmacies and 374 PPMVs were selected from six different states in Nigeria, representing both rural and urban areas, northern and southern regions, and different malaria endemicities. All states of Nigeria were grouped based on malaria endemicity and geopolitical zone and were randomly selected to ensure representation of each. The final six selected states, Kano, Gombe, Kogi, Imo, Edo and Osun were all included in the outlet survey. Outlet owners were interviewed regarding availability of RDTs (defined as having stock available for purchase at the time of the survey) and reasons for stock-outs. In four of the same states, Kano, Gombe, Imo and Edo, 479 households (HHs) were selected and interviewed regarding knowledge and use of malaria diagnostics, healthcare-seeking behaviour and affordability, specifically, how they would diagnose malaria in a family member, whether they had heard of RDTs and ever received a RDT, and whether they thought RDTs were affordable or increase the cost of treatment. Outlets and HHs were selected from one rural local government authority (LGA) and one urban LGA in each state. HHs were also further purposively selected to focus predominantly on low-income HHs and to capture three different monthly income segments in Nigerian Naira (NGN) of: (1) NGN ≤ 18,000, (2) NGN 18,001–36,000, (3) NGN 36,001–100,000. For comparative analysis, a small number of samples from HHs with monthly income NGN > 100,000 were also collected.

Data related to RDTs were analysed using R [13]. Outlet availability of RDTs at the time of the survey was disaggregated by region (north–south), area (urban–rural) and outlet type (PPMV–pharmacy), while HH responses were disaggregated by region (north–south), area (urban–rural) and monthly HH income in NGN. Proportions between sub-groups were compared using a binomial test for difference in proportions or a Chi-square test for trend (for HH income), and p-values significant at the p = 0.05 level are presented.

## Results

### Supply-side—outlet survey

**Fig. 1:**
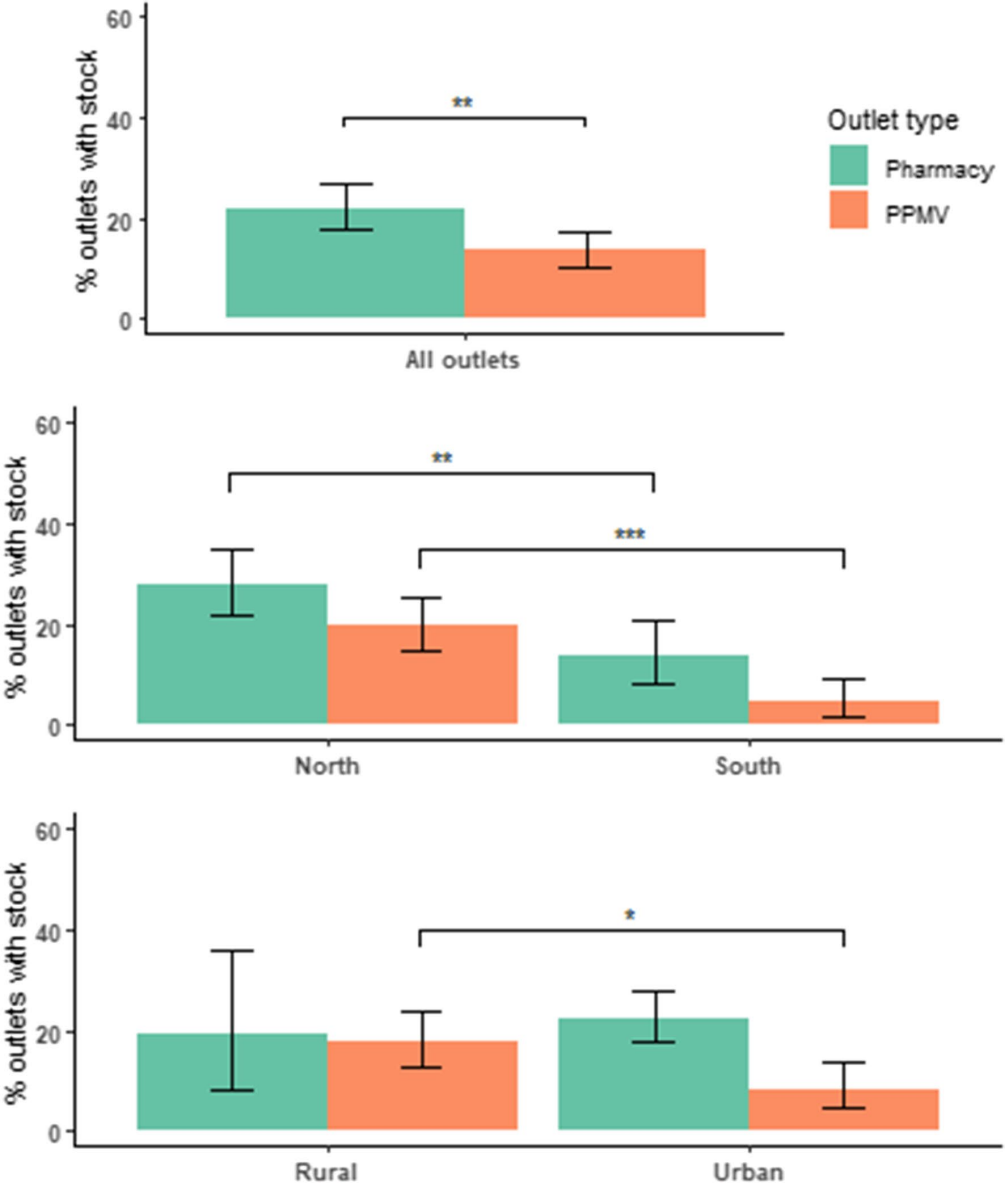
Availability of mRDTs in each outlet type **A** overall, **B** by region and **C** by area. *p = 0.01, **p < 0.01, ***p < 0.001

Availability of RDTs at the time of the survey was low in both outlet types and was significantly lower in PPMVs compared to pharmacies (13.6%, 95% CI [10.3–17.5] versus 22.1%, 95% CI [17.7–27.1], p < 0.01, Fig. 1A). Outlets in the southern states had significantly lower availability of RDTs compared to northern states for both pharmacies (13.6% versus 28.0%, p ≤ 0.01) and PPMVs (4.6% versus 19.7%, p < 0.001, Fig. 1B). Urban PPMVs also had significantly lower availability of RDTs compared to rural PPMVs (8.4% versus 17.9%, p ≤ 0.01, Fig. 1C).

Among outlets that did not have RDTs in stock on the day of the survey (n = 573), 26% said they had previously had stock (at any time). Their reasons for not restocking included low demand (37%), no supply (33%), and low profit margins (3%).

### Demand-side—Household survey

**Fig. 2:**
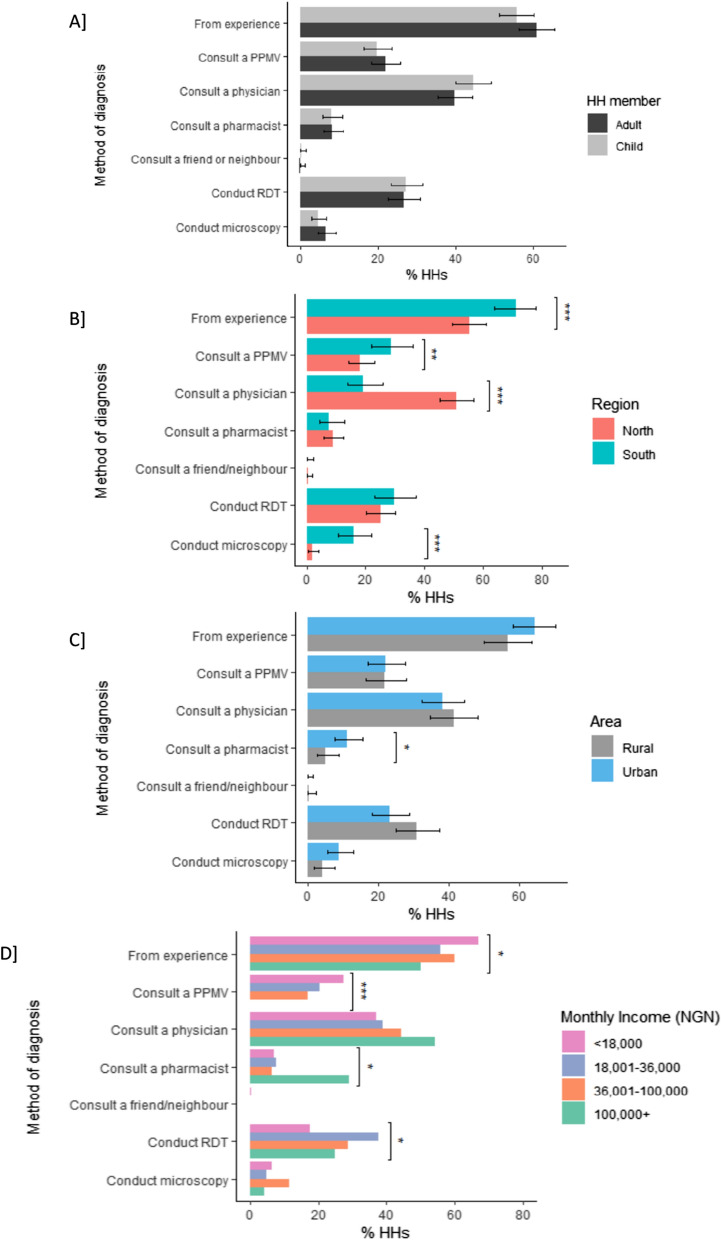
How HH respondents would diagnose malaria for a family member. Multiple responses were available. **A** Respondents stated their preferred method of diagnosis depending on whether the family member was an adult or child. Considering adults only, HH diagnostic behaviour is disaggregated by **B** region, **C** area and **D** HH monthly income. *p ≤ 0.05, **p ≤ 0.01, ***p < 0.001

Most HH respondents reported that they do not follow just one method when asked how they would diagnose malaria for a member of the family (Fig. 2). With multiple responses possible, almost two-thirds of HHs diagnose based on experience. When HHs do seek diagnosis from elsewhere, most go to a public health physician, while 22% of HHs visit a PPMV and 8% visit a pharmacist. One-quarter of HH respondents reported conducting a RDT and a small number reported conducting microscopy. Behaviours were the same if the family member with suspect malaria was an adult or child (Fig. 2A).

By region, HHs in the southern states were significantly more likely to diagnose by experience (p < 0.001), consult a PPMV (p ≤ 0.01) or get a test by microscopy (p < 0.001), while northern states were more likely to visit a public health physician (p < 0.001, Fig. 2B). HHs in urban areas were two-fold more likely to visit a pharmacist than HHs in rural areas (p ≤ 0.05, Fig. 2C). By income level, lower income HHs diagnosed more using experience (p ≤ 0.05) and were more likely to consult a PPMV (p < 0.001), whereas higher income HHs were more likely to visit a pharmacy (p ≤ 0.05, Fig. 2D).

Half of HH respondents had heard of RDTs (48.4%), predominantly from a health institution (58.6%) but some had learned about RDTs from both PPMVs (19.0%) and pharmacies (14.7%). Half of HH respondents also reported having had an RDT in the past (47.4%), mostly from public hospitals (65.6%) or from public health centres (18.9%) and only a few from pharmacies (4.8%) or PPMVs (13.7%).

Most HH respondents did not know about the cost of RDTs and just 38.6% thought RDTs were affordable while a third (33.8%) perceived them as increasing the cost of treating malaria.

## Discussion

Outlets had very low (< 20%) availability of RDTs at the time of the 2018 market survey, particularly urban PPMVs and outlets (both PPMVs and pharmacies) in southern states. Reasons for stock-outs included low demand from consumers and/or supply issues. While the survey did not analyse the supply chain, reports of low consumer demand are supported by the HH survey in which the majority of HHs would diagnose based on experience or visit a public health physician when a HH member had suspect malaria. Despite this, one-third of HHs would visit either a PPMV or pharmacy for malaria diagnosis and the lack of availability of RDTs would have a direct effect on whether these consumers receive the recommended case management [4].

Although about 45% of HHs reported visiting a PPMV/pharmacy for malaria treatment in this same survey as reported elsewhere [5], only 30% reported visiting the same outlets for malaria diagnosis.This is also lower than the proportion of the population that report using these channels as their primary healthcare option in national surveys [2, 7]. The 2018 market survey shows only half of HHs had heard of RDTs and just a third thought they were affordable. Along with lack of supply, poor consumer knowledge and affordability issues may all contribute to the lower proportion of HHs using these same outlets for malaria diagnosis. Among HHs that had heard of RDTs, some had heard of them from PPMVs or pharmacists suggesting these outlets could play a larger role in educating individuals on the role of RDTs, improving consumer demand.

It is not clear why outlets in urban and southern states should have lower availability of RDTs than rural and northern areas. Both urban and southern areas generally have lower rates of poverty (compared to rural and northern areas) and higher consumer demand for treatment from the private sector [5, 14]. However, demand for malaria diagnostics was not as clear-cut, with no difference in demand for urban and rural PPMVs, and high demand for PPMVs but low demand for pharmacies in the south. Diagnosis by experience was significantly higher in southern states, however, and this may contribute at least in part to lower stock in corresponding outlets. Malaria prevalence is higher in the north of the country which may contribute to greater demand and supply of diagnostics to this region, particularly through public health campaigns which have been more targeted to northern regions [2, 5]. Leakage of supplies from the public sector into private channels has been observed [15]. However, northern regions also have more limited access to quality healthcare than those in the south [2]. More enquiry would be needed to explore how issues of poverty, healthcare provision and access and malaria prevalence interact to affect RDT supply and/or cost differences between areas.

The low availability of RDTs sits in stark contrast to the high availability of ACT medicines found by the same survey, whereby > 99% of outlets had stock of any ACT medicine [5]. The AMFm-PSCM period increased supply and affordability of ACT among these same outlets for subsidized and, due to increased market competition, non-subsidized brands [5]. Supply and affordability issues of RDTs could be addressed with a similar scheme thereby creating a more buoyant RDT market. Due to problems with low consumer knowledge of RDTs and presumptive or incorrect treatment provision among providers, any intervention should include behaviour change and educational activities targeted to providers and consumers to promote confidence in tests and the correct course of action given both a positive and negative test result, be it to administer treatment or to refer to another health provider [16, 17]. Conducted in the correct setting and with correct prescribing practices, such a subsidy scheme can be cost-effective [18].

Addressing the deficit in RDT availability may be urgent since the decision from the majority of HHs to diagnose based on experience coupled with low availability of RDTs and high availability of ACT medicines presents a risk for the misuse and overuse of anti-malarials. Such misuse can lead to poor treatment outcomes and adds to drug resistance pressure on the parasite, potentially hindering malaria elimination from the country [19–22]. To date, however, subsidy schemes for RDTs have been few. A 2017 review identified just 12 studies where subsidies had been implemented and these were predominantly small pilots and displayed wide variance in RDT uptake, defined as the proportion of eligible patients for whom an RDT was undertaken; correct ACT provision and adherence to RDT-negative test results [12]. Although important lessons can be learned from these studies, the relevance of training, supervision and retail prices on the outcomes of uptake and adherence were not clear due to contextual and methodological differences. In the Nigerian context, one cluster randomized controlled trial had low RDT uptake of just 8% and poor adherence to test results [23]. A later pilot project conducted in three states of Nigeria showed that efforts to increase availability and affordability of RDTs can increase consumer demand, improve provider profit margins and confidence in diagnostic provision; however, lessons needed to be learned in terms of, for example, supply chain management, action upon negative test results and ensuring stockages of quality assured test kits, as well as addressing leakage of RDTs from the public sector [12, 15, 24]. Given the high prevalence of diagnosis based on experience, an alternative or additional intervention to that of private sector subsidies could be to direct mRDTs for use in self-diagnosis by HH members [25, 26]. Subsidizing ‘bundles’ of RDTs and ACT medicines has also been proposed, whereby a subsidy is only provided for ACT medicines given after a positive RDT result. Such a scheme may improve RDT uptake and correct ACT targeting, however, high confidence in test results is required [27] and there would be challenges in coordinating distribution of both RDTs and ACT medicines to ensure sufficient supply of both. The 2018 Nigerian survey shows the supply of RDTs and ACT medicines has not yet been synchronized and without effective coordination the threat of ACT misuse will remain high. It would be important to determine whether increasing supply of RDTs to private outlets may reduce the use of ACT medicines.

## Data Availability

The datasets used and/or analysed during the current study are available from the corresponding author on reasonable request.

## Abbreviations

ACT: Artemisinin-based combination therapy
AMFm: Affordable Medicines Facility—Malaria (AMFm)
CI: Confidence interval
HH: Household
PPMV: Proprietary and Patent Medicine Vendors
PSCM: Private sector co-payment mechanism
QA: Quality-assured
RDT: Malaria rapid diagnostic test

## References

[1] ACTwatch Group SFH (2015). ACTwatch Study Reference Document: The Federal Republic of Nigeria Outlet Survey 2015.

[2] Nigeria National Population Commission, ICF International. Nigeria demographic and health survey 2018. Abuja, Nigeria and Rockville, Maryland, USA; 2019. https://dhsprogram.com/publications/publication-fr359-dhs-final-reports.cfm.

[3] Nigeria National Population Commission, ICF International. Nigeria demographic and health survey 2013. Abuja, Nigeria and Rockville, Maryland, USA; 2014. https://dhsprogram.com/publications/publication-fr293-dhs-final-reports.cfm?cssearch=342433_1.

[4] WHO (2021). Case management. WHO guidelines for malaria.

[5] Edwards HM, Sarwar R, Mahmud P, Emmanuel S, Maxwell K, Tibenderana JK (2022). The impact of the private sector co-payment mechanism (PSCM) on the private market for ACT in Nigeria: results of the 2018 cross-sectional outlet and household market surveys. Malar J.

[6] Mokuolu OA, Ntadom GN, Ajumobi OO, Alero RA, Wammanda RD, Adedoyin OT (2016). Status of the use and compliance with malaria rapid diagnostic tests in formal private health facilities in Nigeria. Malar J.

[7] National Malaria Elimination Programme Nigeria, Nigeria National Population Commission. Nigeria Malaria Indicator Survey 2015 Final Report. Abuja: Federal Republic of Nigeria; 2016. https://dhsprogram.com/.

[8] Mangham LJ, Cundill B, Ezeoke O, Nwala E, Uzochukwu BSC, Wiseman V (2011). Treatment of uncomplicated malaria at public health facilities and medicine retailers in south-eastern Nigeria. Malar J.

[9] Ansah E, Narh-Bana S, Epokor M, Akanpigbiam S, Quartey A, Gyapong J (2010). Rapid testing for malaria in settings where microscopy is available and peripheral clinics where only presumptive treatment is available: a randomised controlled trial in Ghana. BMJ.

[10] Omache D, Owuor N, Machini B (2020). Predictors of presumptive treatment of uncomplicated malaria among children in private retail outlets in Kenya: mixed effects logistic regression modelling. F1000Res.

[11] Isiguzo C, Anyanti J, Ujuju C, Nwokolo E, de La Cruz A, Schatzkin E (2014). Presumptive treatment of malaria from formal and informal drug vendors in Nigeria. PLoS ONE.

[12] Visser T, Bruxvoort K, Maloney K, Leslie T, Barat LM, Allan R (2017). Introducing malaria rapid diagnostic tests in private medicine retail outlets: a systematic literature review. PLoS ONE.

[13] R Core Team. R: a language and environment for statistical computing. Vienna: R Foundation for Statistical Computing; 2021. https://www.r-project.org/.

[14] National Bureau of Statistics (NBS) (2020). 2019 poverty and inequality in Nigeria: executive summary.

[15] Odugbemi B, Ezeudu C, Ekanem A, Kolawole M, Akanmu I, Olawole A (2018). Private sector malaria RDT initiative in Nigeria: lessons from an end-of-project stakeholder engagement meeting. Malar J.

[16] Prudhomme O’Meara W, Menya D, Laktabai J, Platt A, Saran I, Maffioli E (2018). Improving rational use of ACTs through diagnosis-dependent subsidies: evidence from a cluster-randomized controlled trial in western Kenya. PLoS Med.

[17] Bruxvoort KJ, Leurent B, Chandler CIR, Ansah EK, Baiden F, Björkman A (2017). The impact of introducing malaria rapid diagnostic tests on fever case management: a synthesis of ten studies from the ACT Consortium. Am J Trop Med Hyg.

[18] Bath D, Goodman C, Yeung S (2020). Modelling the cost-effectiveness of introducing subsidised malaria rapid diagnostic tests in the private retail sector in sub-Saharan Africa. BMJ Glob Health.

[19] Nwokolo E, Ujuju C, Anyanti J, Isiguzo C, Udoye I, Bongos-Ikwue E (2018). Misuse of artemisinin combination therapies by clients of medicine retailers suspected to have malaria without prior parasitological confirmation in Nigeria. Int J Health Policy Manag.

[20] Newton PN, Caillet C, Guerin PJ (2016). A link between poor quality antimalarials and malaria drug resistance?. Expert Rev Anti Infect Ther.

[21] Bloland PB (2001). Drug resistance in malaria.

[22] Egwu CO, Obasi NA, Aloke C, Nwafor J, Tsamesidis I, Chukwu J (2022). Impact of drug pressure versus limited access to drug in malaria control: the dilemma. Med (Basel).

[23] Onwujekwe O, Mangham-Jefferies L, Cundill B, Alexander N, Langham J, Ibe O (2015). Effectiveness of provider and community interventions to improve treatment of uncomplicated malaria in Nigeria: a cluster randomized controlled trial. PLoS ONE.

[24] Malaria Consortium. Creating a private sector market for quality-assured RDTs in malaria endemic countries. Project Brief. 2014. https://www.malariaconsortium.org/resources/publications/332/creating-a-private-sector-market-for-quality-assured-rdts-in-malaria-endemic-countries. Accessed 4 Feb 2022.

[25] Douine M, Lambert Y, Galindo MS, Mutricy L, Sanna A, Peterka C (2021). Self-diagnosis and self-treatment of malaria in hard-to-reach and mobile populations of the Amazon: results of Malakit, an international multicentric intervention research project. Lancet Reg Health.

[26] Ranasinghe S, Ansumana R, Lamin JM, Bockarie AS, Bangura U, Buanie JAG (2015). Attitudes toward home-based malaria testing in rural and urban Sierra Leone. Malar J.

[27] Laktabai J, Saran I, Zhou Y, Simmons RA, Turner EL, Visser T (2020). Subsidise the test, the treatment or both? Results of an individually randomised controlled trial of the management of suspected malaria fevers in the retail sector in western Kenya. BMJ Glob Health.

